# Infrared Polaritonic Biosensors Based on Two-Dimensional Materials

**DOI:** 10.3390/molecules26154651

**Published:** 2021-07-31

**Authors:** Guangyu Du, Xiaozhi Bao, Shenghuang Lin, Huan Pang, Shivananju Bannur Nanjunda, Qiaoliang Bao

**Affiliations:** 1 School of Chemistry and Chemical Engineering, Yangzhou University, Yangzhou 225009, China; guangyudu@126.com (G.D.); panghuan@yzu.edu.cn (H.P.); 2 Songshan Lake Materials Laboratory, Dongguan 523808, China; linshenghuang@sslab.org.cn; 3 Joint Key Laboratory of the Ministry of Education, Institute of Applied Physics and Materials Engineering, University of Macau, Macau, China; yb97827@connect.um.edu.mo; 4 Department of Electrical Engineering, Centre of Excellence in Biochemical Sensing and Imaging Technologies (Cen-Bio-SIM), Indian Institute of Technology Madras, Chennai 600036, India; 5 Shenzhen Exciton Science and Technology Ltd., Shenzhen 518052, China

**Keywords:** two-dimensional materials, polariton, mid-infrared, biosensors, medical and healthcare applications

## Abstract

In recent years, polaritons in two-dimensional (2D) materials have gained intensive research interests and significant progress due to their extraordinary properties of light-confinement, tunable carrier concentrations by gating and low loss absorption that leads to long polariton lifetimes. With additional advantages of biocompatibility, label-free, chemical identification of biomolecules through their vibrational fingerprints, graphene and related 2D materials can be adapted as excellent platforms for future polaritonic biosensor applications. Extreme spatial light confinement in 2D materials based polaritons supports atto-molar concentration or single molecule detection. In this article, we will review the state-of-the-art infrared polaritonic-based biosensors. We first discuss the concept of polaritons, then the biosensing properties of polaritons on various 2D materials, then lastly the impending applications and future opportunities of infrared polaritonic biosensors for medical and healthcare applications.

## 1. Introduction

The mid-infrared (mid-IR) is part of the electromagnetic spectrum with the wavelength range of 3–50 μm, in which the unique and strong fundamental movements in terms of rotational and vibrational transitions will take place for many molecules. Mid-IR molecular absorption fingerprint lines are approximately three orders of magnitude higher than those in the near-IR or visible wavelengths, hence it is particularly promising for a wide range of applications in the fields of medicine, basic science, energy, environmental monitoring, and pharmaceutical industry. Moreover, bio-chemical structure analysis and non-intrusive measurement have gained great development with the help of mid-IR spectroscopy [1,2,3,4,5]. For instance, in biological applications, the physical and chemical information of biomolecules samples can be traced out with the powerful tool of “fingerprint” absorption characteristics, which can be employed for biological imaging, drug development and disease diagnosis [6,7,8].

Over the last few decades, researchers have demonstrated various biosensors using noble metal nano-particles (NPs). In comparison, graphene and related 2D materials have attracted significant attention for biosensing applications due to high surface-to-volume ratio, excellent biocompatibility, surface charge, outstanding fluorescence-quenching ability, broadband light absorption, ultrafast carrier mobility, and flexibility [9]. Recently, a surplus of dipole-type polaritonic excitations in 2D materials to enhance the light-matter interactions at sub-wavelength scale has opened up new horizons for mid-IR biosensing [10,11].

Polaritons are hybrid quasiparticles that form because of strong coupling of photons with electric dipoles of the material. There are three types of polariton modes that have been experimentally observed, including exciton-polaritons (EPs), plasmon-polaritons (PPs) and phonon-polaritons (PhPs) [12,13,14,15,16]. Two related length scales are the main figure of merits to define these polariton modes: (i) the extension of the evanescent field in the vertical orientation and (ii) the polariton wavelength propagation through the material-dielectric interface. The incident free-space wavelength is always larger than the polaritons wavelength [17,18]. Surface plasmon-polaritons (SPPs) will be produced when the photon is strongly coupled to the oscillating electrons at the surface of the metal–dielectric interface. Furthermore, at the interface, if an exceedingly large local density of electromagnetic states is presented by the correlatively reduced modal volume, strong electromagnetic oscillations will result in localized surface plasmons (LSPs) [19,20,21,22,23]. Thus, polaritonics afford a way to restrict, manipulate and utilize light at dimensions smaller than the diffraction confinement [24].

The plasmonic biosensing is based on the polaritonic mechanism, where the light confinement and enhancement emerges at the interface between two media with opposite sign permittivity, typically metal and dielectric interfaces, which are highly sensitive to the surrounding refractive index [25,26,27,28,29,30]. Metallic NPs such as gold and silver are the most extensively explored plasmonic materials for biosensing by exploiting the large concentration of electromagnetic energy associated with these optical modes [31,32]. Two-dimensional materials-based polaritons generate even higher levels of field confinement than traditional plasmonic metals and therefore hold great potential for future biosensing applications [33,34].

Taking graphene as an example, extremely confined and electrically tunable plasmon-polaritons were comprehensively studied theoretically and experimentally, opening exciting prospects and providing more opportunities for plasmonic biosensing applications [35]. In addition, a large number of active sites for molecule interactions could be provided by graphene because of its ultrathin structure, large surface area and benzene-ring structure [36,37,38]. Furthermore, considerable flexibility is given to researchers owing to the layer-dependent optical performance of graphene in the exploration of plasmonic biosensing, and also, graphene can play the role of a protective layer for metallic NPs due to its excellent chemical and thermal stability [31,39]. In addition, the satisfactory biocompatibility of graphene further expands its utilization in biosensing fields as well. In this regard, graphene has been deemed as an excellent candidate in the mid- and far-IR ranges for plasmonic biosensors, which overcome the shortcomings of metallic NPs. Recently, sensors based on phonon-polaritons, originating from the interactions between photons and optical phonons, emerge as a new sensing approach by mimicking what has been done on plasmon-polaritons and exhibit its own unique characteristics [40,41,42]. Such polariton-based biosensors can be explored for real-time monitoring of the biomolecular interactions and are highly sensitive to surrounding refractive index changes due to being strongly associated with phonon vibrations. In addition, phonon-polaritons can confine light into extreme sub-wavelength scales and they own longer phonon lifetimes, which is of significant value for the routine biosensing and point-of-care (POC) diagnosis clinical evaluations.

In this review, we first give a brief introduction about the polaritons in 2D materials, including the launching and visualizing of polaritons, various polaritonic modes in 2D materials, the dispersion mechanism of polaritons in graphene and their applications. Thereafter, we discuss two types of biosensors based on three typical 2D materials systems, i.e., plasmon-polaritonic biosensors based on graphene and graphene–metal composites, phonon-polaritonic biosensors based on hexagonal boron nitride (h-BN), and the infrared biosensing mechanisms are illustrated comprehensively. Lastly, we conclude this review by proposing some impending challenges and future opportunities of infrared polaritonic biosensors for further medical and healthcare applications.

## 2. State-of-the-Art Polaritonic in 2D Materials

**Figure 1:**
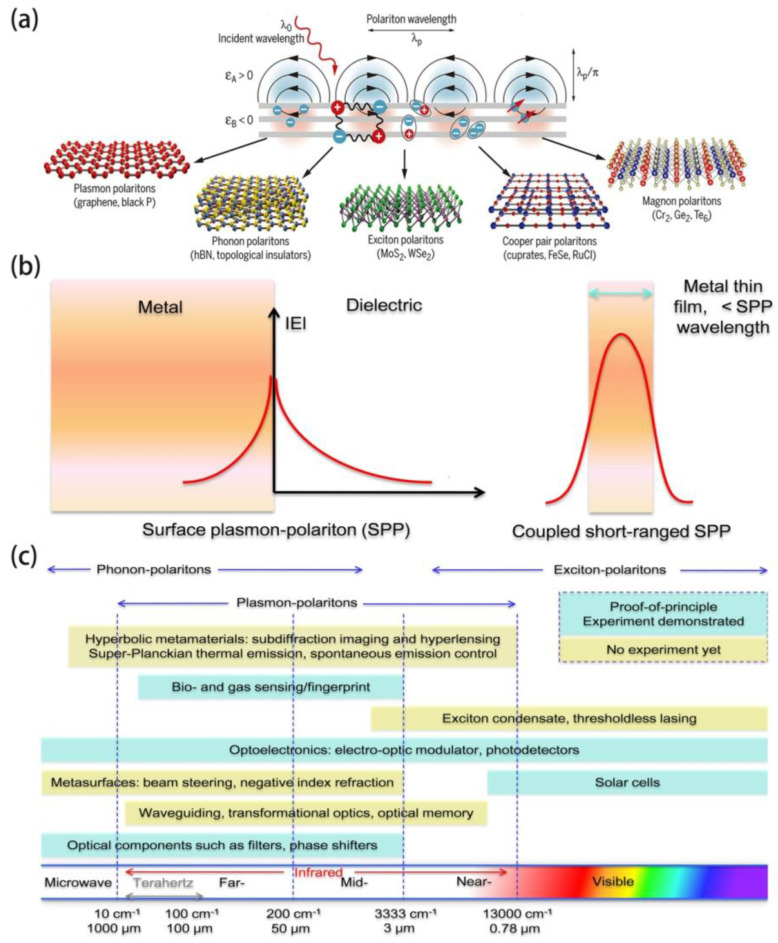
(**a**) Schematic of the formation of polaritons in various vdW materials. (**b**) Schematic explanation of surface plasmon-polaritons in metal and 2D materials. (**c**) Summary of potential applications of polaritons (plasmons, phonons, excitons) in 2D materials and their working frequency ranges. (**a**) Reproduced with permission [43], Copyright 2016, AAAS.

Two-dimensional materials are atomically thin materials in which electrons can move freely in two dimensions and because of quantum confinement the light-matter interactions can be significantly enhanced. The quantum coupling mode of photons with various excited electric dipoles is called polariton. The emergence of 2D materials makes it possible for the polaritonic imaging within atomically thin materials as they provide ideal platform for polaritonic to confine, harness and manipulate light at such dimensions. When illuminated, light is restricted at nanoscale of such materials, much smaller than the incident optical wavelength, demonstrating an enrichment in the correlative electric field strength, which leads to enhanced light-matter interaction [43,44,45,46]. Various hybrid light-matter modes have been proposed depending on oscillations of different charges in 2D materials. As illustrated in Figure 1a, there are various types of polaritons expected in 2D materials. The mostly explored polariton is plasmon-polariton, which is induced by coupling of infrared photons with oscillating electrons in graphene and black phosphorus. Besides this, some other types of polaritons have been studied as well; for example, phonon-polaritons constituted by coupling of infrared photons with atomic vibrations in h-BN and α-MoO_3_, exciton-polaritons in MoS_2_ and WSe_2_, Cooper pair-polaritons in cuprates, FeSe, and RuCl, and magnon-polaritons in Cr_2_, Ge_2_ and Te_6_ ferromagnets [47,48,49,50,51]. These polaritons span wide range of the electromagnetic spectrum, from ultraviolet to microwave.

SPPs are polaritons constituted by electromagnetic modes confined in a plane interface between dielectric and metal (Figure 1b) [13,52,53,54,55,56]. When the film is thin enough, the two surface waves will be coupled in the metal. One scenario is that the longitudinal electric field manifests as an anti-symmetric mode, and the electromagnetic energy is mainly concentrated in the medium on two sides, thus the loss caused by metal is relatively small, and the surface wave can spread for a long distance, which is called ‘long-range SPPs’. Another coupling binding mode is called ‘short-range SPPs’, in which the longitudinal electric field has symmetrical distribution, and electromagnetic energy is mainly distributed in metal, so the loss caused by metal is large and the transmission distance is very short. Compared with isolated SPPs, such ‘short-range SPPs’ are more intensely confined. Over the past few decades, the applications related to metal plasmonic have achieved prominent development in optical biochemical sensing, waveguide and lasing, photovoltaic and photodetection, spectroscopy, and single molecule detection, etc. Nevertheless, the absorption losses and limited operating wavelength (visible and near-infrared) are restricting the progress of metal (e.g., gold and silver) plasmonic. Meanwhile, graphene plasmonic represents a reasonable extension because it provides a new form of 2D-electron gases (2DEG) for plasmons. For instance, parameters in metals that determine the permittivity and the surface plasmon characteristics are stationary, while they can be tunable in graphene by electrical gating or by chemical doping. In addition, the zero-band gap property of graphene signifies that the excitation wavelength ranges from the microwave to the mid-IR regimes, whereas plasmons in simple metal structures are mainly in the visible and near-IR regions due to the optical properties of the noble metals employed. Thus graphene plasmonic based on plasmon-polaritons has been proposed and developed with many unique characteristics that were not observed in noble metals [57,58,59,60].

The graphene plasmonic can be considered as the ultra-short-range SPPs where the light will be constrained to approximately three orders of magnitude smaller than that of the incident free-space optical wavelength. A classical plasmon wave whose wavelength λ_p_ is significantly more than the characteristic length of the electron de Broglie wave λ_F_ should be considered for such model. The oscillation frequency of the plasmon electrons ω_p_ and the phase velocity ν_Ψ_ of the plasmon wave together determine the plasmon wavelength λ_p_, meanwhile, the optical response of graphene can be traced back to its in-plane conductivity σ, which is mainly controlled by electron-hole pair excitations. Therefore, we refer to two equations to explain the graphene plasmonic [43,54].

λ_p_ = 2π/k_p_ = 4π^2^Im{σ/ωε_a_} (1)

where, λ_p_ represents the polariton wavelength, k_p_ means the in-plane polariton wavevector (k_p_ = ω_p_/ν_Ψ_), σ indicates the in-plane conductivity, ω is the incident light frequency, and ε_a_ represents the permittivity of the environment.

The in-plane conductivity (σ) can be represented by Equation (2) as:

σ(ω) = (i/π)·{S_f_/(ω + iτ_f_^−1^)} + (i/π)·{ωS_b_/(ω^2^ − ω_b_^2^ + iτ_f_^−1^)} (2)

where, τ_f_^−1^ is the damping time, S_f_ and S_b_ represent the spectral weight and they can be different in different materials, ω_b_ is the phonon/exciton frequency. The spectral weight S_f_ of graphene can be described as (e/ħ^2^)E_F_, where ħ represents the Planck constant, *e* refers to the charge on the electron and E_F_ is Fermi energy.

Based on these equations, it is clear that the light confinement ratio λ_0_/λ_p_ = (ε_a_/α)(ħ/2E_F_) (λ_0_ is the incident light wavelength) is extraordinarily high in graphene. Furthermore, compared with metal plasmonic, the carrier density of graphene plasmonic can be optically, chemically, and electrically tuned. Graphene plasmonic polaritons operates in the terahertz to mid-infrared frequencies, hence opening its infrared applications, while metal plasmon-polaritons are usually observed in the visible frequencies.

As summarized in Figure 1c, graphene plasmonic polaritons can find promising applications in mid-infrared photonics and optoelectronics, for example, waveguide, filters, optical modulators, photodetection, biochemical sensing and fingerprinting. Recently, there are other newly discovered 2D materials, for example, graphene-dielectric and graphene-metal composites, antimonene, Bi_2_Te_3_, etc., that support similar plasmonic polaritons, which are also potential candidates for these applications [61,62,63,64]. Recently, phonon-polaritonic biosensors have gained much attention since they possess larger quality factors compared with their plasmonic counterparts. In addition, they exhibit extremely long lifetimes, intensive infrared field confinement and flexible tunability via the crystal thickness and the dielectric environment [40,65,66,67]. In the following section, we are going to discuss the state of the art of mid-infrared biosensors based on plasmon and phonon polaritons in 2D materials.

## 3. Infrared Plasmonic Biosensing in Graphene

As compared with traditional noble metal materials, the IR response of graphene is featured by long-lasting SPPs that can be dynamically tuned by electrostatic gating. Additionally, unparalleled spatial confinement of the electromagnetic fields in graphene via SPPs makes it promising for the future integrated mid-IR photonic applications. Notably, the strong confinement of IR light in graphene and tuneability of its SPPs offer great opportunities for mid-IR based light detection, modulation and biochemical sensing applications [68,69].

**Figure 2:**
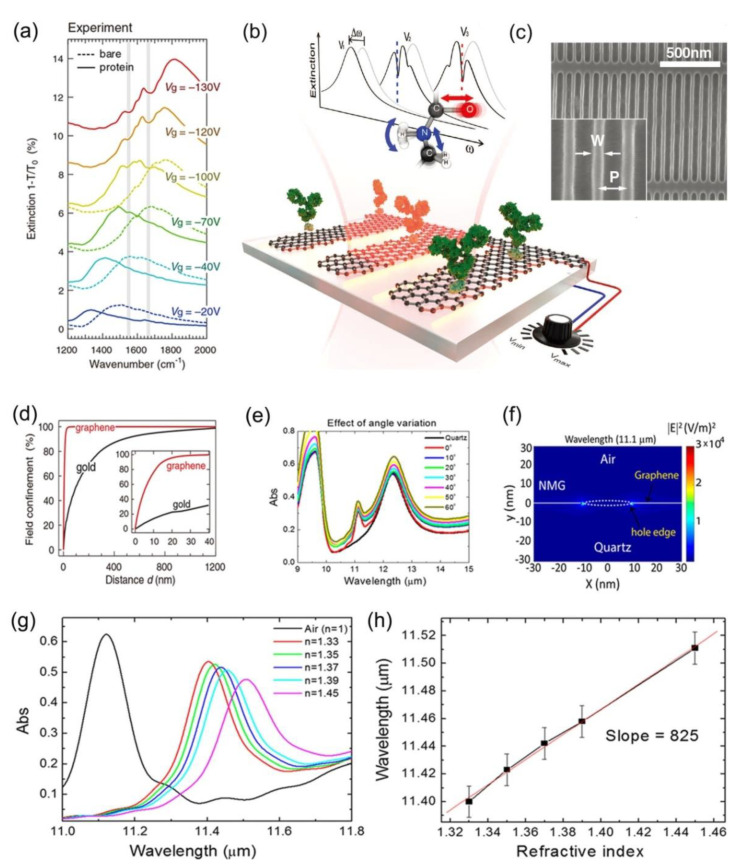
(**a**) Extinction spectra of the graphene nanoribbons for bias voltages Vg from −20 V to −130 V before (dashed curves) and after (solid curves) protein bilayer formation. Gray vertical strips indicate amide I and II vibrational bands of the protein. (**b**) Schematic diagram of the graphene biosensor. (**c**) SEM image of a graphene nanoribbon array (width W = 30 nm, period P = 80nm). (**d**) Percentage of space-integrated near-field intensity confined within a volume extending a distance d outside the nanoantenna. Inset shows a zoom-in ford between 0 and 40 nm. (**e**) Absorption of low-doped NMG on quartz at different incident angles (0–60°). (**f**) |E|^2^ for low-doped NMG at 11.1μm incidence wavelength with an enhanced field maximum value of 36789. (**g**) Absorption of the NMG in vicinity of medium at various refractive indices. (**h**) Resonance wavelength as a function of the refractive index of the surrounding medium, exhibiting a displacement in the resonance wavelength when adding the refractive index of the surrounding medium. (**a**–**d**) Reproduced with permission [33], Copyright 2015, AAAS. (**e**–**h**) Reproduced with permission [70], Copyright 2018, American Chemical Society.

Early in 2015, a new biosensor based on mid-IR plasmonic effect in graphene was proposed. This work opened up new exploration path for biosensing, since it proves the enhanced sensitivity and tunable spectral selectivity of graphene compared with traditional plasmonic metals [33]. More specifically, a highly sensitive tunable plasmonic biosensor for the detection of protein molecules without chemical labeling was demonstrated by utilizing the mid-IR plasmonic response in graphene (see Figure 2a). As shown in Figure 2b, such a biosensor device was fabricated by a graphene layer grown by chemical vapor deposition (CVD) method and patterned on a thin silicon substrate, a plasmon-polariton resonance can be excited in graphene nanoribbons (Figure 2c) when illuminated by a mid-IR beam. The interaction of the mid-IR light beam and protein molecules adsorbed on graphene nanoribbons results in a shift of plasmon-polariton resonance spectrum along with narrow spectral dips related to the specific protein molecular vibrational bands by tuning gate voltage (V_g_). A significant resonance condition related to the localized surface plasmon (LSP) on the graphene nanoribbons can be observed. Even though the thickness of the double layer of protein molecule is only in nanometer scale, the plasmonic resonance frequency shifts to 200 cm^−1^ because of the change in the refractive index at the sensor surface. More importantly, the appearance of two almost undetectable spectral dips at 1550 cm^−1^ and 1660 cm^−1^, which are far away from the plasmonic resonance (e.g., for Vg = −20 V) becomes increasingly intensive with larger spectral overlap (e.g., for Vg = −130 V). The spectral positions of them are consistent with the amide I and amide II bands, respectively, which clearly shows the existence of protein compounds in a label-free chemical specific way. Furthermore, in order to show the advantages of graphene biosensor, metal local surface plasmon resonance (LSPR) biosensor composed of gold dipole nanoantenna is employed for comparison, its noteworthy that by calculating the percentage of near-field strength limited to a given distance from the structure (Figure 2d), 90% of the mode energy is limited to 15 nm on the surface of graphene while the same percentage is distributed within 500 nm from the gold surface, confirming the highly spatial light confinement of graphene in the mid-IR region. Based on the above advantages of the graphene based mid-IR biosensor, it can act as a blueprint or fingerprint for various biochemical molecules.

Later in 2018, nanomembrane graphene (NMG) has also been proposed as an efficient way to develop mid-IR biosensing applications and it has been proved that LSPR can be probed in NMG without the need of external chemical doping, induced potential or even thick reflective metallic layers [70]. The synthesis procedure of NMG is as follows, (i) firstly covering the graphene with Au layer and then annealing to obtain the Au nano-islands, (ii) then the rest of the graphene was protected by Cr layer, and (iii) finally the NMG was fabricated by removing the Au and Cr layers. To confirm whether the NMG plasma is a localized plasmon or a surface wave, the investigation was carried out through the excitation of plasmonic response at all angles of mid-IR incidence. Figure 2e reveals that the absorption peak is reproducible at vertical and oblique incidence from 0 to 60°, confirming that the observed peak is caused by localized plasmons, which can be excited when the momentum mismatch between the incident mid-IR light and the NMG plasmons is broken. By drawing the localization map of electromagnetic field distribution in NMG at incidence 11.1 μm resonance wavelength, it is authenticated that the plasmonic response is localized at the edge of NMG, and the field enhancement factor is found to be 184 (Figure 2f). For mid-IR biosensor application, finite-difference time-domain (FDTD) was employed theoretically, analyzing the resonance shift caused by the refractive index change of the medium around the NMG. The variation of absorption wavelength as a function of refractive indices (n) is shown in Figure 2g,h demonstrates that the relationship between refractive index and wavelength is linear with a sensitivity of 825 nm/RIU where the dynamic range is 0.12 RIU, indicating its potential for highly sensitive mid-IR biosensors.

## 4. Infrared Plasmonic Biosensing in Hybrid Graphene-Dielectric System

Inspired by works mentioned above, many studies about graphene for plasmonic biosensing have been carried out. However, large momentum mismatch between plasmons and photons has always impeded direct light absorption in graphene, which limits its potential in mid-IR biosensing application.

**Figure 3:**
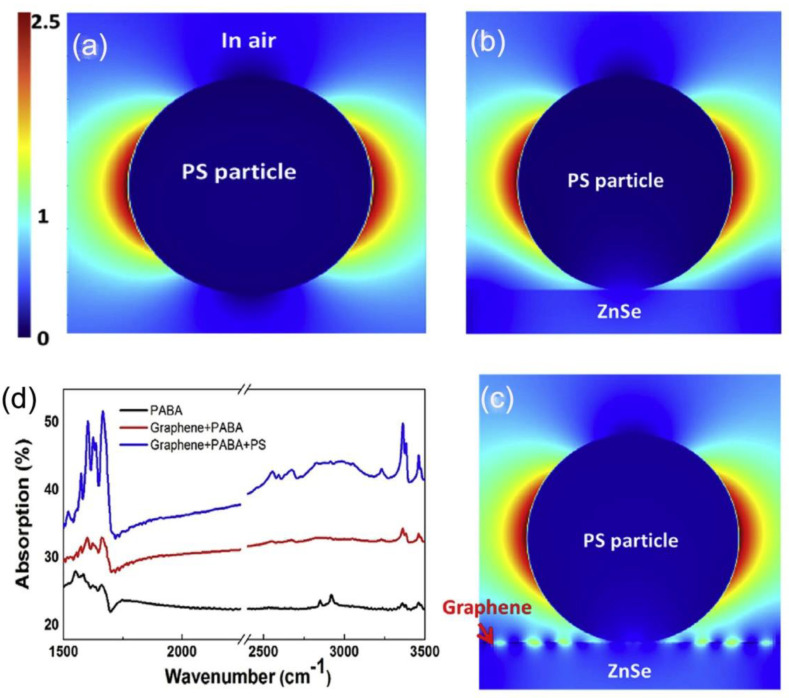
(**a**–**c**) Electricfield images surrounding PS particles with an excitation wavelength of 5460 nm, for PS particle in air (**a**), on a ZnSe substrate (**b**), and on graphene/ZnSe (**c**), respectively. (**d**) Mid-IR absorption spectrum for a higher density of PABA on ZnSe (black), graphene/ZnSe (red) and graphene/ZnSe/PS (blue). (**a**–**d**) Reproduced with permission [71], Copyright 2017, Pergamon-Elsevier Science Ltd.

To overcome the extremely large momentum mismatch between incident photons and plasmon polaritons, many approaches have been proposed to enhance the IR light absorption in terms of coupling efficiency. For instance, one can introduce dielectric beads onto graphene to tackle this problem [71]. More specifically, the SPPs are excited by the interference of scattering waves from the polystyrene (PS) bead, which propagate several microns in graphene, boosting the interaction between mid-IR light beam and bio-molecules, and thus enhancing the sensitivity. The electric field optical confinement with PS bead in air, ZnSe, and ZnSe/graphene substrates are presented in Figure 3a–c, respectively. Interestingly, when the PS bead is coated on the ZnSe/graphene substrate, the electric field in graphene oscillates with the enhancement of electric field intensity can be observed evidently, indicating that the PS bead can excite the SPPs on graphene. As illustrated in Figure 3d, graphene/PS system exhibits more profound mid-IR absorption for testing para-aminobenzoic acid (PABA) compared to pure graphene.

## 5. Infrared Plasmonic Biosensing in Hybrid Graphene-Metal System

Many reports have introduced the employment of only graphene or metal to excite SPPs to strengthen the mid-IR based biomolecular fingerprint [72]. There is a limitation in measuring the binding of low molecular weight analysts in the mid-IR range. Therefore, a hybrid system consisting of graphene and metal has been proposed to enhance the limit of detection [64]. Such hybrid systems can offer some distinctive advantages over single material-based optical or electrical biosensor devices [73,74,75]. Firstly, the hybrid sensor allows high sensitivity detection of small molecular weight analytes. Secondly, the resonance displacement caused by the change in graphene–metal optical conductivity is a clear indication of variation of carrier density due to the molecular binding. Finally, the hybrid sensor shows excellent consistency and stability due to its insensitive to the reduction of mobility of graphene carriers [76,77,78].

**Figure 4:**
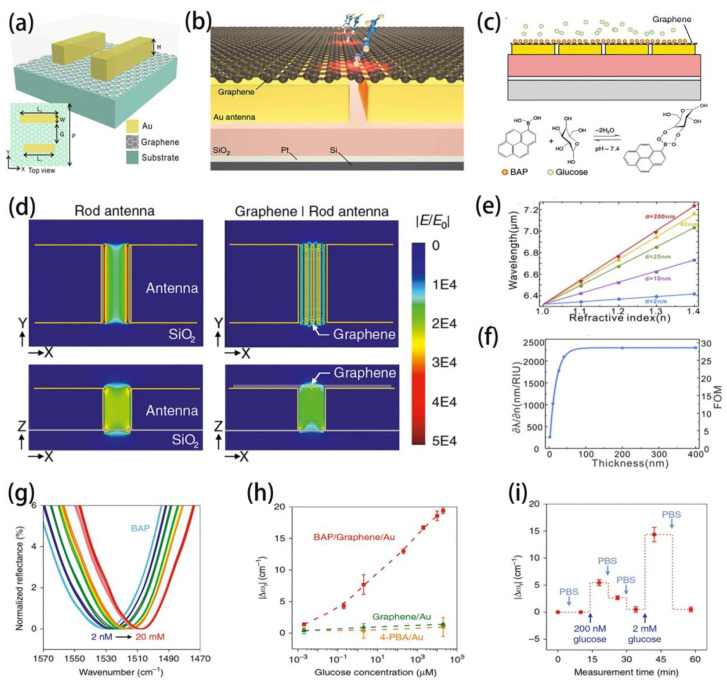
(**a**) Schematic diagram of a hybrid graphene/Au nanoantenna composition and the linearly polarized waves propagating at normal incidence in a Cartesian coordinate system. (**b**) Schematic diagram of graphene–metal metasurface, exhibiting small molecules are adsorbed on the suspended graphene. (**c**) Schematic diagram of a hybrid graphene–metallic biosensor. (**d**) Near-field images for rod antenna. Left: Bare Au nanoantennas. Right: Graphene-coated Au nanoantennas. The graphene carrier density is 8 × 10^12^ cm^−2^. (**e**) Changes of transmittance angles as a function of refractive index for various thickness from 2 nm to 200 nm. (**f**) Calculated FOM with different thickness. (**g**) Spectral measurement of glucose from 2 nM to 20 mM on the biosensor surface. (**h**) Plasmonic resonance |Δω_r_| as a function of glucose concentration in various structure of biosensors. (**i**) Reversible nature of plasmonic resonance |Δω_r_| with different glucose concentrations. (**a**,**e**,**f**) Reproduced with permission [81], Copyright 2019, Optical Soc Amer. (**b**–**d**,**g**–**i**) Reproduced with permission [79], Copyright 2018, Nature Publishing Group.

Based on the advantages mentioned above, recently, an unique biosensor has been proposed based on a hybrid metal nanoantenna array and single-layer graphene (Figure 4a,b) [79]. In this work, glucose concentrations were measured to prove the sensitive quantification of small molecular biomarkers with a hybrid system (Figure 4c). The near-field optical images for naked Au nanoantenna and graphene-coated Au nanoantenna are compared in Figure 4d. It is shown that the electric field distribution changes significantly after adding a single layer of graphene to the Au nanoantenna, and the SPPs is excited on the hybrid meta-surface, which offers more opportunities for the measurement of biochemical molecules at very low concentrations. The Au nanoantenna as coated with single layer of graphene sheet is surrounded by a cover layer with a thickness of d and refractive index of n. One of the highlights of this work lies in demonstrating high sensitivity of such sensor for detecting ultra-thin cover layer down to nanometric size in mid-IR range. As shown in Figure 4e, the sensitivity (slope curves) reduces with the decrease of cover layer thickness (d); however, the sensitivity can be maintained as high as 1025 nm/RIU even if the thickness reduces to 10 nm. This lower limit of detection is achieved due to the exceedingly high spatial confinement of graphene SPPs in the near-IR field. Additionally, the figure of merit (FOM) of different cover layer thicknesses is calculated in Figure 4f, the surface sensitivity (S) is 2304.40 nm/RIU with a saturation value (FOM) of ∼28.81 for thickness larger than 120 nm. The curve indicates that such sensor can still work in mid-IR range even if the thickness decreases to only few nanometers scale. Based on this work, a fano-resonance hybrid graphene/metal sensor was also demonstrated [80], of which the resonance intensity can be strengthened by adding the graphene layers, exhibiting a FOM of approximately 158.7 and an outstanding sensitivity of about 7.93 µm/RIU. Treating such hybrid graphene-Au nanoantenna as a biosensor with boronic acid-pyrene (BAP) and exposing it to glucose solutions, the plasmonic resonance (ω_r_) shifted to lower wavenumbers and demonstrated a detection range from 2 nM to 20 mM (Figure 4g). The plasmonic resonance (ω_r_) is shifted due to the boric acid with an empty orbital extract electron from graphene resulting in p-doped graphene initially, and this reaction is weakened when combined with glucose molecules. The plasmonic resonance (ω_r_) shift for various configurations (BAP/Graphene/Au, Graphene/Au, and 4-PBA/Au) as a function of glucose concentration are illustrated in Figure 4h, which clearly indicates the hybrid graphene/metal system with BAP surface functionalized exhibits superior sensitivity and a lower limit of detection. Furthermore, the switching response of plasmonic resonance |∆ω_r_| were observed when the biosensor was washed with fresh buffer solution and exposing it to the glucose with high concentration, indicating that such biosensor could detect the decrease and increase of glucose concentration due to the reversibility of glucose boric acid binding (Figure 4i). These results indicate a new approach to achieve high-performance plasmonic biosensing based on graphene-metal hybrid system.

## 6. Novel 2D Antimonene for Plasmonic Biosensing

2D antimonene exfoliated from Sb has superior physicochemical performance than other typical 2D materials [82]. When compared with graphene, antimonene has an analogous sp^2^-bonded honeycomb lattice, better hydrophilicity, excellent stability and stronger spin-orbit coupling. Additionally, the special antioxidant capacity of antimonene plays a pivotal role in biomolecules detection. Based on these properties, 2D antimonene materials have already been applied in field effect transistors (FET), thermophotovoltaic (TPV) cells, photothermal therapy (PTT) and nonlinear optics. Furthermore, suitable band gap, high surface-to-volume ratio, excellent biocompatibility, high carrier mobility, and excellent stability make the 2D antimonene extremely suitable for biosensing applications.

**Figure 5:**
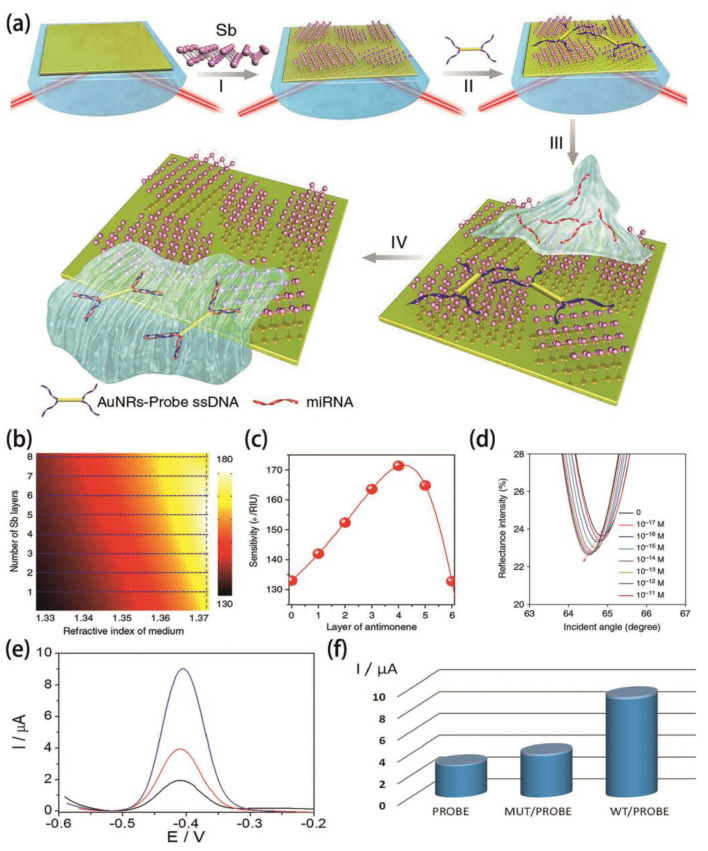
(**a**) Fabrication procedure of the antimonene biosensor. I. The antimonene nanosheets were assembled on the surface of Au film. II. AuNR-ss DNAs were adsorbed on the antimonene nanosheets. III. miRNA solution with different concentrations flowed through the antimonene surface and paired up to form a double-strand with complementary AuNR-ssDNA. IV. The interaction between miRNA with AuNR-ssDNA results in release of the AuNR-ssDNA from the 2D antimonene, the reduction in the molecular of the AuNR-ssDNA on the SPR surface results in significant decrease of the SPR angle. (**b**,**c**) The changes in the sensitivity with respect to the different number of antimonene layers when the refractive index of the sensing medium is 1.37+∆n in such biosensors. (**d**) SPR spectra with miRNA-21 concentrations ranging from 10^−17^ to 10^−11^ M. (**e**) Antimonene/gold screen-printed electrodes modified with the DNA probe before (black line) and after hybridization with the MUT (red line) or WT (blue line) form from clinical samples. (**f**) Bar diagrams of the direct DNA sensor response to clinical samples. (**a**–**d**) Reproduced with permission [63], copyright 2019, Nature Publishing Group. (**e**,**f**) Reproduced with permission [83], Copyright 2020, American Chemical Society.

Various expression levels can be detected by microRNA (miRNA) in cancer, which can have an influence on metastasis, carcinogenesis and cellular transformation. Fluorescence methods with dye molecule labels are widely utilized for miRNA detection. Label-free quantification of miRNA in molecular level remains to be developed. To meet this challenge, a unique surface plasmon resonance (SPR) biosensor based on 2D antimonene has been proposed [63]. Figure 5a shows the steps involved in fabrication of 2D antimonene coated SPR biosensor and miRNA detection. The SPR signal from the sensor is extremely sensitive to variations of analyte refractive index, which can determine the quantity of miRNA. In addition, the sensitivity can be tuned with the number of antimonene layers and the refractive index of the medium as shown in Figure 5b,c. It can be observed that the highest sensitivity is achieved at 4 antimonene layers. The target miRNA-21 was detected at a relative low concentration, and the SPR responses are shown in Figure 5c. We can clearly observe the left-shift in the SPR angles caused by the hybridization of miRNA-21 even at a very low concentration (10^−17^ M). These results show that the combination of 2D antimonene and SPR structure provides an ultra-sensitive detection method for miRNA and manifests as a new way for early diagnosis, staging and monitoring of cancer.

Following this research work, 2D antimonene has also been explored in DNA sensing [83]. Nowadays, a common method to detect mutations is to employ the classic Sanger strategy for DNA sequencing. However, these conventional diagnostic tools are very expensive and involve strenuous process. Therefore, the development of a fast and affordable DNA biosensor is of great significance. In this work, differential pulse voltammetry (DPV) curves and a bar diagram of the biosensor signal for the wild-type (WT) analyte (used as a control) and mutated (MUT) DNA samples are exhibited in Figure 5e,f, respectively. The data show that when hybridization takes place in the form of MUT, the current intensity produced is less than half of that observed in the form of WT, indicating the high sensitivity of such biosensor for detecting DNA mutations.

Based on the research works mentioned above, recently, a MXene/antimonene composite based SPR biosensor with an adhesive TiO_2_ layer has been reported [61]. The sensitivity of such biosensor with and without TiO_2_ layer are 224.26°/RIU and 178.76°/RIU, respectively. Here, TiO_2_ acts as a thin oxide layer with high refractive index, it can combine with plasmonic metal materials to form bimetallic layer and enhance SPR signal. These research works pave new way for exploring more antimonene-based composites in biosensing applications.

## 7. Infrared Phonon Polaritonic Biosensing in h-BN

**Figure 6:**
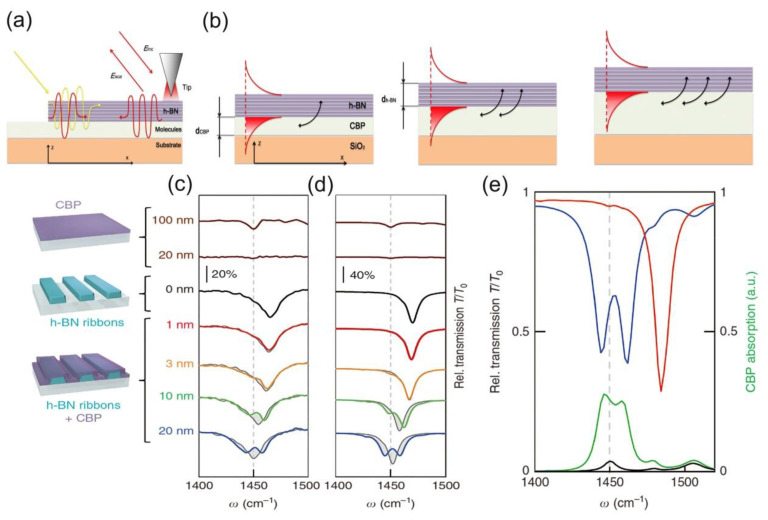
(**a**) Demonstration of the phonon-polariton based biosensor experiment. E_sca_ and E_inc_ represent the tip scattered radiation and electric fields of incident. (**b**) Schematic diagram of phonon-polaritons on different thickness of CBP layers. The red line represents the phonon-polaritons field, while the red enveloped zone indicates the overlap between the CBP molecules and the phonon-polaritons field, which enhances with the addition of CBP thickness, strengthening the coupling between molecules and phonon-polaritons, as shown by black arrows. (**c**,**d**) Infrared transmission spectra of h-BN nanoribbons with various thick d_CBP_ layers covering. (**c**) Experimental transmission spectra of a 20 × 20 μm^2^ size h-BN nanoribbon with ribbon width of w = 158 nm and period of D = 400 nm. (**d**) Simulative transmission spectra for a bare h-BN nanoribbon (black curve, w = 167 nm, D = 400 nm), a CBP-coated one (same color in c) and for a bare CBP layer (brown curves). (**e**) Numerical research of strong coupling between CBP molecular vibrations and HPhPs. (**c**–**e**) Reproduced with permission [40], copyright 2018, Nature Publishing Group.

Recently, researchers have explored phonon-polaritonic-based biosensors considering that phonon polariton has a larger quality factor Q, which is the physical quantity that represents the damping property of the oscillator [84,85]. The production of superior infrared phonon-polaritonic biosensors are enabled with the emergence of van der Waals (vdW) materials such as h-BN [86,87,88]. Especially, highly confined hyperbolic phonon polaritons (HPhPs) in h-BN vdW materials have attracted more attention due to outstanding light guiding ability at the nano-size. Surface-enhanced infrared absorption (SEIRA) spectroscopy was employed for detecting tiny organic molecules by h-BN infrared phonon-polaritonic biosensors [40]. Such biosensor device is composed of an array of h-BN nanoribbons covered with 4,4-bis (*N*-carbazolyl)-1,1-biphenyl (CBP) on a CaF_2_ substrate. The CBP molecules were chosen for their distinct vibrational modes in the h-BN nanoribbons. Additionally, CBP can be uniformly deposited by simple thermal evaporation, and the thickness of the layer can be adjusted at sub-nanometer scale. After the device fabrication, real-space observation has also been carried out by using scattering-type scanning near-field optical microscopy (s-SNOM) to launch phonon-polaritons in a h-BN layer above CBP (Figure 6a,b) [89]. Interestingly, fringes emerge at the molecular vibrational resonance frequency, revealing strong interaction between CBP molecular vibrations and phonon polaritons in h-BN samples. In addition, the fringe oscillation signal caused by phonon polariton interference can be observed in a Fourier transform infrared (FTIR) spectroscopy image. Furthermore, it was found that the mode splitting Ω (the minimum vertical separation between true frequencies of quasi normal modes) and the coupling power between CBP and h-BN are enhanced with the adding CBP thickness because of more phonon polariton field coupling in CBP molecule layer (Figure 6b).

To observe surface-enhanced infrared absorption, infrared transmission spectra was taken as shown in Figure 6c. It was noteworthy here that a displacement of hyperbolic phonon polariton resonance dip at almost full width of half maximum along with significant shape variety is observed on a 20-nm-thick CBP layer covered nanoribbon (blue curve), which is attributed to the strong coupling of phonon polariton and CBP resonance caused by intense localized fields in h-BN. Furthermore, the influence of the CBP layer thickness has also been investigated, i.e., the alignment of hyperbolic phonon polaritons resonance dip begins to shift in a 3 nm thick layer (yellow curve) and become more obvious in 10 nm thick layer (green curve). Such influence is verified by full-wave electromagnetic simulations in Figure 6d. Moreover, strong coupling between molecular vibrations and hyperbolic phonon polaritons gain a further verification via numerical simulations of the transmission spectra as shown in Figure 6e. Particularly, the green curve (Figure 6e) which represents the absorption of the CBP molecules is achieved by integrating the volume of the entire layer and directly proves this effect. This work has demonstrated novel opportunities for further development in infrared phonon-polaritonic biosensing.

Following this research, a special type of phonon-polaritonic biosensor based on single isotope ^10^B h-BN was proposed, and its infrared response were studied [67]. Compared with natural h-BN, the lifetime of single isotope ^10^B h-BN biosensor device is longer due to the reduction of photon scattering from randomly distributed isotopes, as well as the increase of mass factor by 50% on average, which enables the detection of nanoscale molecules by monitoring the refractive index and surface enhanced absorption spectroscopy. In addition, the strong coupling between phonon polarized exciton resonance and molecule vibrations can even be realized in the device. This research work provides a new blueprint in the field of quantum optical chemistry and selective catalysis with chemical modification at nanoscale.

## 8. Conclusions and Outlooks

Polaritons in 2D materials open new horizons for investigating lattice dynamics and opto-electronic properties because they support the highest confinement degree of various polaritonic modes among all known materials. Especially, advanced near-field imaging techniques such as s-SNOM and nano-FTIR enable polaritons to be launched and their propagation and confinement visualized at nanoscale in 2D materials. The obtained polaritonic images allow us to explore the dispersion relation regions of various excitations which surpass the reach of traditional optical techniques. Furthermore, sub-nanoscale concentration of electromagnetic energy can be exploited based on these polaritonic modes and superior field confinement can be achieved in 2D materials in comparison to conventional plasmonic metals. These properties make 2D materials promising for IR biosensing applications and provide unique opportunities in developing future diagnostic technique. Label-free detection is a critical superiority of polaritonic-based biosensing, nevertheless, samples with high purity are needed to avoid non-specific interactions, which may degrade the polaritonic signal. To achieve real-time monitoring of biomolecules, the addition of microfluidic channels are considered as promising candidates to both purify and deliver the samples. However, there are some limitations; for instance, the challenge of locating target molecules at sensor hot spots, the specificity of recognizing analyte, the intrinsic absorption loss of plasma metals at various optical frequencies, and the challenge to set nanoparticles in the user-defined region of microfluidic channels. Additionally, the properties of polaritonic biosensing is restricted by noise because of the quantum performance of light, which is called the shot-noise limit (SNL), which comes from the Heisenberg uncertainty principle [90]. Enhancing field intensities is one of the most common strategies to strengthen polaritonic biosensing properties, yet this method seems to be not practicable due to the photodamage of biological samples. To overcome these problems, using phase change instead of reflection intensity, or using quantum light source instead of classical light source can be applicable.

Moreover, from the perspective of materials, it is still challenging to obtain large-area and high-quality single-layer 2D materials. Among the various synthesis strategies, CVD is the most outstanding way to solve this problem, especially in the fabrication of graphene and its various composites. The size and quality of 2D materials depend largely on the precursor, substrate, pressure, and temperature kept in the CVD growth process. It is necessary to carefully choice these parameters for mass fabrication and controllable synthesis of high-quality 2D materials for biosensing applications. Additionally, decorating 2D materials with plasmonic metal NPs or depositing them on the substrate, improves excellent interface contact and is favorable to energy transfer, charge shift, plasmon coupling, and ultimately improves the sensing properties and enhance sensitivity. vdW force and electrostatic interaction are two main routes to combine 2D materials and the substrates, resulting in inferior stability of the device. By contrast, 2D materials with a large number of free radicals can covalently combine with the matrix and metal NPs. Hence, it is of great significance to treat the surface grafted free radicals of 2D materials for the preparation of polaritonic biosensors.

In the future, more efforts are expected to explore novel biosensors based on phonon-polaritons because of their long polaritonic mode lifetimes and slow speeds, which are especially advantageous for enhancing local emitters, molecular vibrational modes, and strengthening applications requiring strong light-matter interactions. Besides, exciton-based polaritonic biosensors may be exploited for their long-lived feature which enables further observation of their condensation and superfluidity at relatively high temperatures. Furthermore, the combination of 2D metamaterials and quantum plasmonics has great potential to provide enhancing phase-change sensitivity for improving limit of detection.
